# Improving practice guidelines for the treatment of denture-related erythematous stomatitis: a study protocol for a randomized controlled trial

**DOI:** 10.1186/s13063-017-1947-y

**Published:** 2017-05-05

**Authors:** Raphael F. de Souza, Muhammad Faheem Khiyani, Carolina A. L. Chaves, Jocelyne Feine, Jean Barbeau, Ramón Fuentes, Eduardo Borie, Luciana C. Crizostomo, Claudia H. Silva-Lovato, Pierre Rompre, Elham Emami

**Affiliations:** 1 Division of Oral Health and Society, Faculty of Dentistry, McGill University, Montreal, QC Canada; 2 Department of Dental Materials and Prosthetics, School of Dentistry of Ribeirão Preto, University of São Paulo, Ribeirão Preto, SP Brazil; 3 Faculty of Dental Medicine, Université de Montréal, Montreal, QC Canada; 4 Research Centre in Dental Sciences (CICO), Faculty of Dentistry, Universidad de la Frontera, Temuco, Chile; 5 School of Public Health, Public Health Institute, University of Montréal’s Hospitals Research Center, Université de Montréal, C.P. 6128, succursale centre-ville, Montréal, QC H3C 3J7 Canada

**Keywords:** Denture stomatitis, Complete denture, *Candida*, Oral candidosis, Pragmatic trials

## Abstract

**Background:**

Denture-related erythematous stomatitis (DES) is a chronic biofilm-mediated disease, affecting one in every three complete denture wearers. Antifungals are the treatment most commonly prescribed by oral health professionals, based on the belief that colonization by *Candida* spp. is the main cause of DES. However, high recurrence rates and adverse effects are commonly observed, prompting the need for practice guidelines regarding treatment. Results from our pilot study demonstrate that palatal brushing can reduce the palatal inflammation and potentially associated *Candida* carriage without any need for antifungal therapy. The objective of this study is to validate these pilot results by means of a randomized controlled trial (RCT) and provide a practice guideline for clinicians.

**Methods/design:**

A pragmatic, two-parallel-arm, multicenter RCT will be conducted in Canada, Brazil, and Chile. Fifty-two adult complete denture wearers presenting with moderate to severe DES will be allocated randomly to two groups: the Intervention arm will consist of palatal brushing and standard oral and denture hygiene measures, while the Control arm will include only standard oral and denture hygiene measures. The study outcome will be the oral *Candida* carriage. Participants will be assessed at baseline, and at 3 and 6 months post intervention. Descriptive, bivariate, and mixed models with repeated measures will be performed following the intention-to-treat principle.

**Discussion:**

This pragmatic RCT will serve to provide a clinical practice guideline regarding the use of preventive measures in the treatment of biofilm-mediated oral diseases. Moreover, it will have a great impact on reducing the harm of antifungal overtreatment on patients suffering from DES.

**Trial registration:**

ClinicalTrials.gov, NCT02686632. Registered on 15 February 2016.

**Electronic supplementary material:**

The online version of this article (doi:10.1186/s13063-017-1947-y) contains supplementary material, which is available to authorized users.

## Background

Denture biofilm is a complex layer of microorganisms, including bacteria and yeasts, embedded in an extracellular polysaccharide matrix [1, 2]. Toxins and metabolic waste produced by these microorganisms cause cell injury and inflammation [3, 4]. Evidence suggests that microorganisms commonly found in oral biofilms can play a role in the development of systemic conditions through various pathways of infection and inflammation [5–8]. Thus, maintaining the oral microbial ecological equilibrium is critical because it plays a significant role in the prevention of oral diseases and their associated systemic complications [9]. However, many oral biofilm-mediated diseases continue to be treated based on outdated practice guidelines established with weak supporting evidence.

Practice guideline development is a complex process that is impeded by a lack of sound research and scientific evidence. This makes clinical decision-making a challenging task, potentially resulting in adverse consequences for patients, including misdiagnosis, maltreatment, overprescription of medications, unwanted side effects and harm, as well as depletion of resources of the health care system [10–13]. In turn, these consequences may even lead to legal issues for healthcare providers.

Denture-related erythematous stomatitis (DES) is an example of a biofilm-mediated disease treated according to suboptimal practice guidelines. DES is a longstanding and recurrent inflammatory condition, mostly observed on the palatal mucosa and easily diagnosed during routine oral examination. Similar to other types of inflammation, it results from the host’s protective cell response to traumatic, chemical, or microbial cell injury, ensuing vasodilatation, increased microvascular permeability, and tissue edema [14, 15]. While often asymptomatic, some patients may present with mucosal tenderness and bleeding, halitosis, burning sensation, xerostomia, and dysphagia [16, 17]. DES is the most prevalent oral disease and the main indicator of poor oral health among the completely edentate population, affecting one in every three individuals wearing removable dental prostheses [18–22]. A higher prevalence of DES is seen in older people, due to long-term denture use, lack of dexterity in performing oral hygiene, polymedication, and decreased host immunity [23, 24]. However, children and adults wearing acrylic partial dentures, obturator prostheses, and ortho-appliances can also be affected by DES [25–27].

Because complete tooth loss can nowadays be considered a disease of poverty, DES is mostly seen in individuals with low socioeconomic status, who are less likely to visit health care professionals on a regular basis [22, 25, 28]. This may put this population at risk because studies have shown a link between this oral pathology and serious systemic diseases such as bacterial endocarditis and aspiration pneumonia. This is critical for hospitalized patients, individuals with a compromised immune system, and older people with cognitive impairments and dementia [5, 7, 29, 30].

Although the etiology of DES is still undiscovered, the literature at large suggests that this disease is multifactorial, with colonization by *Candida albicans* being the main risk factor [31]. Therefore, clinicians usually prescribe antifungals along with oral hygiene instructions and prosthesis adjustments [32–34]. However, a high recurrence rate and recolonization by *Candida* after cessation of antifungal therapy are widely reported [35–37]. Retreatment with antifungals is therefore common. This unjustified use of antifungal medication may expose patients to adverse effects over time, most notably medication resistance [38]. A recent meta-analysis on this topic showed the inefficacy of antifungals in the treatment of denture stomatitis [39]. Furthermore, several research findings question the etiological role of *Candida* spp. in the initiation of inflammation and suggest that candidal colonization may be secondary to tissue injury and inflammation [40, 41].

Current evidence highlights the need for providing new treatment guidelines for DES. Recent findings from the Emami et al. research group showed that brushing the palate is a promising alternative to antifungal medication [41]. However, these encouraging pilot results should be validated by a randomized controlled trial. A recent systematic review of different treatment methods for DES also highlighted the need for a randomized trial on soft tissue hygiene [42]. Therefore, the objective of this pragmatic clinical trial is to provide evidence on the effectiveness of palatal brushing for the treatment of DES. The knowledge gained from this study will help in developing clinical guidelines aimed at promoting the oral health of edentate individuals.

### Study hypothesis

Edentate individuals with DES using palatal brushing and standard oral hygiene measures after each meal over a period of 6 months will show a clinically meaningful lower level of oral *Candida* carriage when compared with those who use only standard oral hygiene measures.

## Methods/design

### Design

**Fig. 1:**
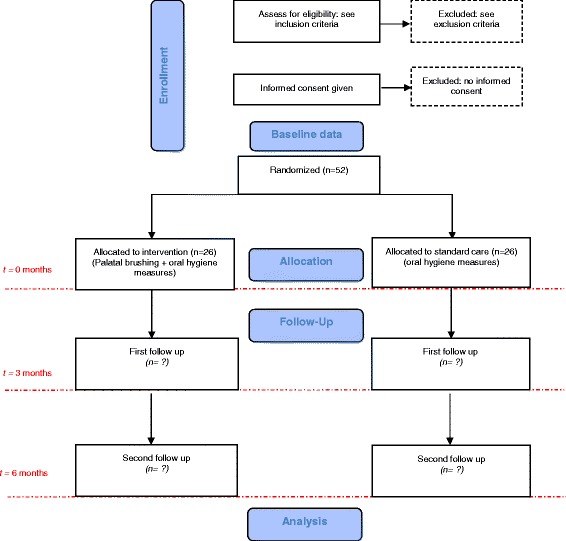
CONSORT study flowchart. Numbers of withdrawn and lost participants will be reported for each follow-up

**Fig. 2:**
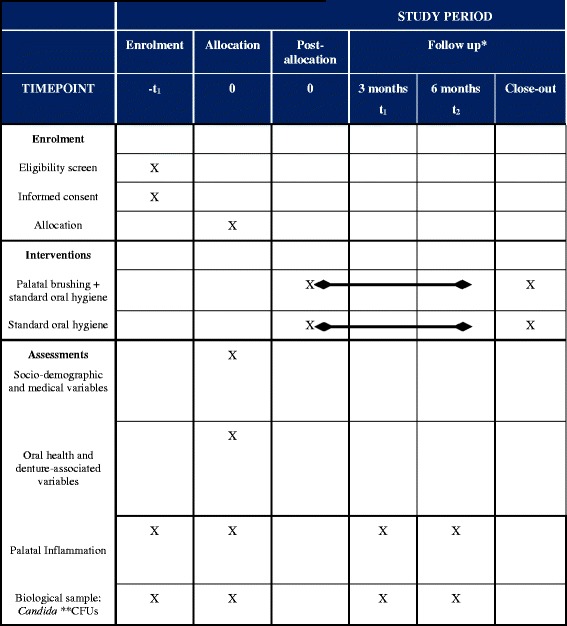
Study schedule: enrolment, allocation, interventions, baseline, and post-intervention assessments

The trial will be a single-blind, parallel group, multicenter randomized controlled trial (Fig. 1). Figure 2 shows the study timeline, according to the Standard Protocol Items: Recommendations for Interventional Trials (SPIRIT) diagram. Additional file 1 presents the SPIRIT checklist.

### Setting and location

The study will be conducted at the following four locations:

1. Coordinating centers: Faculty of Dentistry, Université de Montréal and Faculty of Dentistry, McGill University, Montreal, Canada.
2. School of Dentistry of Ribeirão Preto, University of São Paulo, Ribeirão Preto, Brazil.
3. School of Dentistry, Universidad de La Frontera, Temuco, Chile.

Activities in all centers will involve at least two team members who will work directly with participants: a research trainee and a faculty researcher. These members will hold a DDS or equivalent title.

### Participants’ eligibility criteria

#### Inclusion criteria

Exclusion criteria include: (1) providing consent prior to participation in the study; (2) being at least 18 years of age; (3) wearing a maxillary complete conventional denture; and (4) having moderate to severe signs of DES. Clinical diagnosis of DES will be conducted using the Schwartz index [43], as previously reported in the pilot phase of the trial [41].

#### Exclusion criteria

Exclusion criteria will be: (1) having oral mucosal lesions apart from DES; (2) having systemic conditions which predispose to *Candida* spp. infection, such as uncontrolled diabetes; (3) having a history of chemotherapy/radiotherapy; (4) having taken or used antibiotics, steroidal agents, or antifungal agents in the 4 weeks prior to the study; (5) being scheduled to replace existing dentures with new ones during the period of the trial; and (6) already using palatal brushing as a routine oral hygiene procedure.

### Planned trial interventions

#### Intervention

Palatal brushing and standard oral/denture hygiene measures.

#### Comparison

Standard oral/denture hygiene measures.

Palatal brushing will be performed as follows: (a) removal of dentures from the oral cavity; and (b) gently brushing the palate using a soft-bristle toothbrush (Oral-B CrossAction Pro-Health; Procter & Gamble, Iowa, IA, USA) for approximately 1 minute without any toothpaste.

Standard oral/denture hygiene measures will be performed as follows: participants will be requested to clean their dentures by: (a) washing the dentures thoroughly in tap water and making sure that there is no visible debris on the internal and external surfaces; (b) placing 2 cm of toothpaste on a denture brush (Oral-B Denture Brush; Procter & Gamble) and brushing internal and external surfaces for approximately 2 minutes; and (c) rinsing thoroughly with tap water.

Participants in both study arms will be required to perform the prescribed procedures after each meal while continuing with their routine denture-wearing habits for the duration of the trial.

### Participant recruitment

Study participants will be recruited through local newspapers and information brochures distributed in dental clinics, dental schools, and residential homes for older people. Respondents at each center will be asked to contact a dedicated telephone number with a voicemail. The research trainees will return the calls to describe the study and to assess major inclusion criteria. Potentially eligible participants will be invited to visit the responsible research center(s) in their home country. During this visit, they will meet with a study coordinator or responsible research trainee who will explain the trial in further detail and assess the candidate’s eligibility criteria. If the candidate does not meet the eligibility criteria and is not enrolled in the study, the reason for exclusion will be recorded.

Eligible candidates will be invited to participate in the study and given sufficient time to read the informed consent and ask any questions pertaining to their participation. After signing the consent form (Additional file 2), the participants will be formally enrolled in the study and baseline assessment will be conducted.

### Randomization, blinding, and allocation

After baseline assessment, participants will be allocated randomly to each study arm. Off-site computer-assisted, centralized stratified block randomization will be conducted. Stratification will be conducted according to trial centers. Each participant will receive a sequentially numbered, sealed, opaque, and tamper-proof envelope from the trial coordinator at the respective center, containing the concealed allocated intervention. The trial coordinator will give each participant a demonstration as well as verbal and written instructions for following the protocol in their corresponding study arm.

This person will also serve as the contact person for the participants to discuss any issues regarding the study treatments throughout the duration of the trial. The research trainee and all other investigators involved in the study will be blinded to the intervention.

Because of the nature of the intervention, it is not possible to blind the participants. However, all appointments will be organized in such a way as to minimize communication between participants and to prevent potential study contamination. Additionally, participants will be requested to keep their group allocation confidential and not discuss it with the research team involved in the study analysis. The intervention will start the day after the study participants receive the opaque envelopes allocating them to one of the groups.

### Study outcome

The study outcome will be the decrease in oral *Candida* carriage defined by *Candida* spp. colony forming unit (CFU) counts, originating from both denture biofilm and palatal mucosa.

### Explanatory variables

Data on a series of potential confounders and/or modifiers of study outcome will be collected. These will include baseline data on the following.

#### Sociodemographic and medical variables

Age, sex, ethnicity, education, medical history, medication profile, and smoking habits will be assessed at baseline.

#### Oral health and denture-associated variables

The baseline data will include dental history, years of edentulism, history of tooth loss, age of the maxillary dentures, and oral hygiene/denture hygiene habits (cleaning frequency, nocturnal wear, and mouthwash use), as well as satisfaction with oral health and existing dental prostheses. Denture cleanliness will be evaluated using the modified Hoad-Reddick classification [44, 45]. A clinical assessment of vertical dimension of occlusion, stability, and retention of the maxillary prosthesis, as well as shape and resiliency of the maxillary edentulous ridge, will be carried out according to standard prosthodontic criteria [44].

The data on oral hygiene/denture hygiene habits and denture cleanliness will be collected at each follow-up to create a time-dependent hygiene variable.

### Data collection

Data collection will be conducted using a self-administered questionnaire and a clinical examination conducted by trained and calibrated dentists using a front surface mirror and probe (XP23/QW; Hu-Friedy, Chicago, IL, USA). Photographs of the palate will be taken with a Nikon D90 camera (105 mm f/2.8 D, macro flash SB-21; Nikon Co., Tokyo, Japan). These photographs will be used to obtain a diagnostic consensus from research team members.

To facilitate conducting the study, baseline data will be collected at the participant’s first visit to the research center, following informed consent. However, if the candidates need more time to reflect on their participation and commitment to the study, another visit will be scheduled for baseline assessment. Follow-up data collection will be conducted at 3 and 6 months post randomization by the same examiner at each research center. The data collection sessions are estimated to last approximately 45–60 minutes.

### Biological sample collection and analysis

Collection of denture biofilm will be performed following the standardized sonication technique protocol [44, 46, 47]. Palatal biofilm will be collected using a sterile swab on the central palatal mucosa (1 cm^2^) [48], placed in a sterile tube with 5 ml saline, and sonicated for 2 minutes. In order to standardize the collection time, samples will be collected between 9 a.m. and 12 p.m. The biological samples will be immediately transferred to the microbiological laboratory at each center.

Swabs and sonicates will be mixed separately with saline, diluted according to the defined protocol [44], inoculated on Sabouraud-Dextrose 4% agar for *Candida* spp., and incubated at 37 °C for 48 hours. Identification of different *Candida* species will be confirmed by growth on selective culture medium (CHROMagar Candida, Paris, France). CFUs will be counted and expressed as CFU/ml, after correction for volume and dilution factor.

### Sample size

Sample size estimation assumes the minimal clinically important between-group difference in the *Candida* CFU/ml mean change score to be 30,000, with a standard deviation of the distribution of the change score of 31.6 (based on estimates from our pilot data) [41]. These parameters lead to a total sample size of 52 participants (26 for each study arm) to ensure a power of 85% for rejecting the null hypothesis with a conservative dropout rate of 10%.

### Statistical analysis

Data entry and analysis will be conducted in a blinded fashion. An interim analysis has not been planned due to the low frequency of adverse effects observed during the pilot trial [41]. Basic descriptive statistical analyses will be performed for all variables after being tested for normality.

Bivariate analysis, as well as mixed-model analyses for repeated measures, with time and intervention as independent variables, will be conducted following testing for normality. In the case of non-normal distribution, the Brunner Langer nonparametric test will be used.

All analysis will be performed in adherence to the intention-to-treat principle [49]. *p* ≤ 0.05 will be considered statistically significant. All analysis will be performed using SPSS version 24 and SAS 9.4 for Windows.

### Data management, monitoring, and auditing

All data collected and entered at each center will be periodically checked by a data monitoring committee composed of two independent researchers and one end-user representative. Additionally, an independent audit could be conducted by the ethics committee of each center at any time.

### Risks, participant safety, and trial adherence

In the pilot phase of this study it was observed that for some participants palatal brushing caused minor discomfort; specifically, mild pain and minor bleeding of the mucosa occurred in 40% of the 48 study participants in the first few days [41]. However, all participants were able to adhere to the study protocol and instructions, and there were no dropouts.

Based on these results, we expect a high degree of compliance. Furthermore, in order to enable participants to follow the recommended protocol, they will be provided with written instructions on the protocol. Participants will also receive telephone calls periodically throughout the length of the trial, to reinforce their trial adherence.

All adverse effects observed will be recorded at each follow-up. If the researchers detect any incidental finding (e.g., a mass or lesion) at any time throughout the duration of the study, the participant will be informed and subsequently referred to the appropriate health professionals.

### Confidentiality

All data related to each participant will receive a code and will be kept strictly confidential. Information linking the participants’ identities to the codes will be kept in a password-protected file and computer.

### Dissemination and knowledge transfer

The objective of the knowledge transfer activity for this study would be to disseminate results to patients in order to improve oral and denture hygiene practices and to recommend treatment guidelines for clinicians to follow. To this end, a summary of the study results will be shared with the study participants via email, and with the general public through social media and the Web. Furthermore, data and the results from the research will also be published in international peer-reviewed journals. The results will also be presented at national and international professional research conferences targeted at both general practitioners and prosthodontists. Efforts will be made to seek funding opportunities to organize workshops/seminars at universities to inform educators and instructors of the future generation of dentists, concerning new practice guidelines for DES.

No identifiable information will be disclosed through these means of results dissemination. Palatal photographs of the participants could be presented for scientific purposes, but these images do not disclose identity.

## Discussion

Current treatment practices for DES lack evidence from good quality randomized controlled trials and fail to justify the rationale for the repeated use of antifungals. In contrast, using oral hygiene measures like palatal brushing may have the potential to provide a conservative, yet clinically and economically effective alternative for the long-term treatment and/or prevention of DES.

For this randomized controlled trial, we are using a pragmatic approach in order to study a diverse sample population of complete denture wearers with DES, belonging to varied socioeconomic, cultural, and educational backgrounds, recruited through multiple international centers. This approach, therefore, will ensure maximum generalizability of our results, which in turn will be more meaningful for patients and clinicians alike.

The information from this trial will be used to address the gaps in practice guidelines for clinicians and to improve the standard of care for millions of edentate people wearing dental prostheses. The information from this study will also improve clinical decision-making and potentially protect edentate patients from harm caused by ineffective treatment.

### Trial status

Recruiting since April 2016 (Brazil and Chile) and October 2016 (Canada).

## Additional files

Additional file 1: Presents the SPIRIT 2013 checklist. (DOCX 53 kb)

Additional file 2: Presents the consent form in English. (DOCX 52 kb)

Additional file 3: Presents the funding documentation. (PDF 77 kb)

Additional file 4: Presents the ethical approval documents in English. (PDF 476 kb)

## Abbreviations

CFU: Colony forming unit
DES: Denture erythematous stomatitis
