# Supplementary material for: Improving practice guidelines for the treatment of denture-related erythematous stomatitis: a study protocol for a randomized controlled trial
Source: Trials. 2017 May 5;18:211. doi: 10.1186/s13063-017-1947-y (PMC5420092; doi:10.1186/s13063-017-1947-y)
Supplement: Supplementary file 2 — Presents the consent form in English. (DOCX 52 kb) [file 13063_2017_1947_MOESM2_ESM.docx]

**Efficacy of palatal brushing in patients with denture stomatitis: A randomized controlled trial.**

**Student responsible**

Dr Faheem Khiyani, DDS, MDPH, candidate for Ph.D in Sciences Biomédicales

Faculté de médecine, Université de Montréal

Laboratoire de recherche en santé orale et réhabilitation bucco-faciale

C.P.6128 succursale centre-ville

Montréal, H3C 3J7

Tel: (514) 923-9270

Email: muhammad.faheem.khiyani@umontréal.ca

**Research director**

Dr Elham Emami, DDS, MSc, PhD

Faculté de Médecine dentaire, Université de Montréal

C.P. 6128, succursale centre-ville

Montréal, H3C 3J7

Tel: 514-343-6053

Email: elham.emami@umontréal.ca

**Principal investigator at McGill University**

Dr. Raphael F de Souza, DDS, MSc, PhD

Faculty of Dentistry, McGill University

2001 McGill College, suite 534
Montreal, Quebec H3A 1G1

Tel:(514) 398-4777 ext. 00052

Email: raphael.desouza@mcgill.ca

**Research co-directors**

Dr. Jocelyne Feine, DDS, MS, HDR, FCAHS, FITI

Faculty of Dentistry, McGill University

2001 McGill College, suite 500
Montreal, Quebec H3A 1G1

Tel:(514) 398-7203

Email: jocelyne.feine@mcgill.ca

Dr Jean Barbeau, BSc (U laval), PhD (U laval)

Faculté de médecine dentaire, Université de Montréal

C.P. 6128 Succursale Centre-Ville

Montréal, H3C 3J7

Tel: (514) 343-2366

Email: jean.barbeau@umontréal.ca

# INFORMATION AND CONSENT FORM

**General Information**You are invited to take part in this research project because you wear a complete upper denture and you have on the palate an inflammation called ‘*denture stomatitis*’.

Before deciding whether or not to participate in this project, it is important that you understand the purpose of this study and its progression. This information and consent form explains the goals, procedures, risks and benefits of this study. The contact information of the people involved in this project are listed in case you need to contact them. You must understand the nature of the project to make an informed choice.

This form may contain words that you do not understand. We invite you to ask any questions that you have to the researchers and other staff involved in this project and ask them to explain any words or information that is unclear.

Please carefully read the information below and listen to the explanations given by the researcher. Take your time to read and make your decision. If you decide to participate in the study, you must sign and date this consent form and a copy will be given to you.

**Description of the research project**

Denture stomatitis is an inflammatory condition of the palate in people who wear complete denture. It is usually treated with antimicrobials or disinfectants that might have side effects and that are usually no longer effective after the treatment is stopped.

Our previous studies have shown that brushing the palate may reduce the extent of inflammation. This study could therefore, confirm this finding and enable dentists to treat patients with denture stomatitis in a more conservative, simple and economical way.

The objective of this study, which will include approximately 180 participants, is to obtain scientific evidence on the effect of palatal brushing in the treatment of denture stomatitis. In addition, this clinical trial will compare palatal brushing to standard oral hygiene measures at a 6 month follow-up. The results will allow us to recommend guidelines for the treatment and prevention of denture stomatitis.

In this study, we will determine the extent of the inflammation of your palate and the number of yeast cells attached to your dentures and to your palate during each visit. We will then be able to compare the results obtained at the first visit with those obtained after 3 months and 6 months of palatal brushing.

# You are eligible to participate in this study if:

# You are 18 years of age or older

# You wear a complete upper denture

# And you are affected by moderate to severe types of denture stomatitis.

- You are not eligible to participate in this study if:

1. You have diabetes or xerostomia, if you are immuno-compromised, or if you are treated with chemotherapy or radiotherapy
2. You have received antibiotics, corticosteroids or antifungal agents in the last 4 weeks prior to the study
3. You have a mild form of denture stomatitis.
4. Brushing your palate is part of your normal oral care.
5. You will change your existing denture during the period of the study.

**Nature of participation and duration of the study**

If you volunteer to participate in this study and sign the consent form, a dentist-researcher will:
• Complete a questionnaire with you during your first visit that includes socio-demographic information, aspects of your lifestyle and a short dental and medical history.

• Examine your mouth and, especially, your palate.

• Examine your dentures.

• Take photographs of your palate.

• Take a sample of saliva from your palate with a Q-tip swab and collect a sample of your saliva.

• Take your upper denture for about 15 minutes: it will be placed in a sterile plastic bag with saline and will be cleaned in an ultrasonic bath for 5 minutes. This bath is done inside a device that removes microbes and food particles by using sound waves, without damaging your dentures.

• These procedures will be repeated after 3 and 6 months.

The dentist-researcher will give you a manual toothbrush with soft bristles and give you instructions about its use. You should brush your palate with this brush after meals and before sleeping for 6 months.
You will also be asked to maintain your usual oral and denture hygiene routine.

A second invitation will be done to collect saliva, in order to check the severity of inflammation in your mouth. This is as an optional part of this study thus, if you accept, we will provide another consent form. After consent, we will ask you to drool inside a tube during few minutes. Collected saliva will be used to quantify proteins linked to inflammation; no genetic test will be performed. This part will be done before you start using your new brush and after 6 months.

The table below shows the stages of the study:

| Visit | Stages of the study | Treatments / data collection | duration (hours) |
| --- | --- | --- | --- |
| 1 | First examination (0 months) | Consent form, questionnaire, oral examination, photographs and sampling | 1,5 |
| 2 | Second examination (3 months) | Oral examination, photographs and sampling | 1 |
| 3 | Third examination (6 months) | Oral examination, photographs and sampling | 1 |

**Associated risks and inconveniences**

An examination of the palate, palatal swab and saliva collection usually do not cause any risk, pain or discomfort to the patient. Your denture will be soaked in saline and cleaned with ultrasound; these do not compromise the material of the denture, nor its color and texture. The time devoted to the study and travel to/from the clinic are the main inconveniences associated with participating in this research project.

During the first few days of palatal brushing, some patients may have minor discomfort, like mild pain or minimal bleeding of the mucosa at the site where the palate was brushed. Throughout the duration of the project, if anything of concern is detected in your mouth, you will be informed. In such a case, the research team will provide a letter of reference for you to seek appropriate medical advice.

The optional testing of saliva will only lead to the quantity of few specific proteins linked with inflammation and will not disclose other information, e.g. if you have any infectious disease. If we decide to make another analysis or to store it for future studies, we will ask your consent before.

**Benefits**

You may or may not benefit personally from taking part in this project. One potential benefit is a possible reduction in the inflammation of your palate. You will also receive the required brushes free of charge. You will benefit from having your dentures cleaned with ultrasound technology at each study visit. This study will provide researchers with new information about the impact of palatal brushing in denture wearers, and the results of this study may help in the development of better treatment for denture stomatitis.

**Financial Compensation**

As a compensation for your costs to travel to/from the clinic, you will receive an amount of $25 at each visit, for a total of $75 for 3 visits.

**Protection of confidentiality:**

During your participation in this project, the research team will gather information about you in a dossier that is required to meet the project’s scientific objectives.

All data relating to your participation in this study will be kept strictly confidential and will be protected with a code. The key code connecting your name to your research file will be kept by the researcher responsible. Depending on the location where you come for the 1^st^ study visit, research data will be kept in: (1) the research laboratory of Dr. Emami, Director of this study, at the Université de Montréal, or (2) the office of Dr. de Souza, principal investigator for McGill University. The data will be kept secured for 25 years after the end of the study and will then be destroyed.

Similarly, a code will also be given to your saliva sample that will link it to your name. To ensure the confidentiality of this code and to prevent the disclosure of your name or identity, only the study staff will take note of this code. The name that corresponds to the code will be kept in a password protected computer file in the research laboratory of Dr. Emami at the Université de Montréal, Roger-Gaudry Building, room D523. Only authorized members of the research team will have access to it. The saliva samples will be stored for a maximum for 25 years after which they will be destroyed.

Members of the McGill Institutional Review Board, or persons designated by this Board, may access the study data to verify the ethical conduct of this study.

**Communication of results**

Once the study is completed, we plan to inform the study participants and the general public of the results of the study, in order to improve oral and denture hygiene practices. Thus, a summary of the study results will be shared with you via email if you wish, or conventional mail if you do not use or do not have access to email or internet. The results will also be available to you and to the general public through social media and the web.

The results of this research will be presented at scientific meetings and published in scientific journals. No identifiable information will be disclosed through these methods. The photographs of your palate could be presented for scientific purposes, but they will not show your identity.

**Voluntary participation and right of withdrawal**

Your participation is voluntary and, by signing this consent form, you are not waiving any of your rights. In addition, you do not liberate the researchers of their legal and professional liability.

You may withdraw from this study at any time, without giving any reason. You simply have to notify the contact person in the research team, and this can be done verbally or in writing. In case of withdrawal, the information that had been collected up until the time of your withdrawal will not be destroyed so as not to jeopardize the integrity of the study.

Throughout the study, you will be notified of any new information which might make you reconsider your participation in the study.

Your participation, refusal to participate or withdrawal from the study will have no impact on the care that you receive at the dental clinics of the Université de Montréal and McGill University.

**Discontinuation of the study**

The researcher in charge of the research project or the Research Ethics Committee may terminate your participation, without your consent, if new findings or information indicates that your participation in the project is not in your interest, if you do not follow the instructions of the research project or if there are administrative reasons to abandon the project.

**Contact information for questions**

1. Questions about the study:

For more information about this research or if you want to withdraw from the study, you can contact the researcher director at the University of Montreal, Dr. Elham Emami, by telephone at (514) 343-6053 or by email at: [elham.emami@umontreal.ca](mailto:elham.emami@umontreal.ca).

You can also contact the student responsible, Dr. Faheem Khiyani by telephone at (514) 923-9270 or by email at: [muhammad.faheem.khiyani@umontreal.ca](mailto:muhammad.faheem.khiyani@umontreal.ca).

1. Questions about your rights and complaints:

For more information on ethical conditions concerning your participation in this project, please contact the Research Ethics Advisor by email: ceres@[umontreal](mailto:ceres@umontreal.ca).ca or by telephone at (514) 343-6111 ext. 2604.

For more information about your rights as participants, please visit the portal of the participants at the Université de Montréal at the following address: <http://recherche.umontreal.ca/participants>.

Any complaints about your participation in this research project may be addressed to: (1) the Ombudsman at the Université de Montréal, by telephone (514) 343-2100 or by e-mail at [ombudsman@umontreal.ca](mailto:ombudsman@umontreal.ca); or (2) Mrs. Ilde Lepore, the Ethics Officer at McGill University, by telephone (514) 398-8302 or by e-mail at [ilde.lepore@mcgill.ca](mailto:ilde.lepore@mcgill.ca). These reference people accept collect calls. Both speak French and English and takes calls between 9 and 17h.

**CONSENT FORM:**

The research project described in this consent form has been explained to me. I am aware of the purpose of this study, what I am asked to do, and the risks and benefits of taking part. Any questions I had about this study have been answered, and I can obtain more information at any time during the study. I am aware that I can withdraw from this study at any time. I consent to take part in this study. I do not give up any of my legal rights by signing this consent form. I will receive a copy of this consent form for my records.

|  |  |  |
| --- | --- | --- |
| Name of participant  (printed) |  | Signature of participant |
|  |  | Date : |

Conservation of contact information (optional)

I agree that my contact information is kept and that I could be contacted to participate in another research project.  Yes  No

**Commitment and signature of the research student:**
I, Faheem Khiyani, declare to have explained the purpose, nature, benefits and risks of the study, and to have answered all the questions of the participant. The research team and I agree to comply with what was agreed to in this information and consent form. A signed copy of this document has been given to the participant.

Signature ……………………………………………. Date………………………………......................
