# Supplementary material for: Improving practice guidelines for the treatment of denture-related erythematous stomatitis: a study protocol for a randomized controlled trial
Source: Trials. 2017 May 5;18:211. doi: 10.1186/s13063-017-1947-y (PMC5420092; doi:10.1186/s13063-017-1947-y)
Supplement: Supplementary file 3 — Presents the funding documentation. (PDF 77 kb) [file 13063_2017_1947_MOESM3_ESM.pdf]

Canadian Institutes of Health Research | Instituts de recherche en santé du Canada

AUTHORIZATION FOR FUNDING

CIHR (Canadian Institutes of Health Research) has approved funding as detailed below. Subject to the approbation of funding by Parliament, these funds will be made available to the business officer at the indicated institution for disbursement.

AUTORISATION DE FINANCEMENT

IRSC (Instituts de recherche en santé du Canada) vous accorde les fonds tel qu'indiqué ci-dessous. Suivant l'affectation des crédits par le Parlement du Canada, les fonds seront mis à la disposition du trésorier de l'établissement indiqué qui s'occupera des versements.

201410MC2-336308-CL-CFCA-165458

06/03/2015

Institution Paid/Établissement chargé d'administrer les fonds: Université de Montréal

Recipient(s)/Bénéficiaire(s): EMAMI, Elham
Restorative Dentistry
Faculty of Dentistry
Université de Montréal

Program/Programme: CIHR Clinician Scientist - Phase 2
Award Accepted Renewal

Primary Institute/Institut principal: Musculoskeletal Health and Arthritis

Project Title/Titre du projet:

Fostering Oral Health Through Interdisciplinary Research: Intervention, Access and Impact

PAYMENT DETAILS/DÉTAILS DES VERSEMENTS — Funding Reference Number/ No. de Référence du financement: **MC2 — 122248**

Period / Période	Type	Amount by Type / Montant par type	Total by Fiscal Year / Total par exercice	
01/04/2015 to 31/03/2016	Salary	$60,000	$60,000	2015-16
01/04/2016 to 31/03/2017	Salary	$60,000	$60,000	2016-17
01/04/2017 to 31/03/2018	Salary	$60,000	$60,000	2017-18

Progress Report Required: / Rapport des progrès réalisés requis: **Not Applicable**

Application to Renew Funding Required: / Demande de renouvellement des fonds requis: **Non-Renewable**

NOTES:

This award is for three (3) years and is not renewable.

The above salary contribution includes the employer's share of fringe benefits.

You received this funding because your colleagues volunteered their time to assist CIHR with the review of your application. We ask that, as a recipient of CIHR funding, you will participate in CIHR peer review activities if invited.

By drawing on the funds provided through this award you agree to the terms and conditions set out in the attached "Conditions of Funding", any breach of which may result in CIHR taking remedial action as described therein. CIHR requires that its contribution to your research personnel award be acknowledged in all written and oral presentations of your research results, including scientific articles, news releases, news conferences, public lectures and media interviews.

Jennifer Lee

Jennifer Lee
Acting Deputy Director, Open Programs
Research, Knowledge Translation and Ethics Portfolio

cc
___ Supervisor/Directeur
___ Dean/Doyen
___ Host/Hôte
___ Administration
___ Accountant/Comptable
___ CIHR Finance/Service des Finance d'IRSC
___ Other/Autre

Canada

McGill

Financial Services
Research **F**inancial **M**anagement **S**ervices (RFMS)
3465 Durocher Street, Room 321
Montreal, Quebec H2X 0A8

New Fund Notice

November 29, 2016

Dear Dr. / Prof. de Souza,

Please be informed that a new fund has been opened per the award parameters and Sponsor's governing terms and conditions as outlined by the Office of Sponsored Research (OSR) approval summary provided to you via email communication. Below you will find your new fund code along with other related information. **Note: Budgets for US and Foreign grants/contracts will be converted to CAD currency at the award approval date and recalculated for subsequent installments(renewals).**

FUND INFORMATION	CONTACTS
**Fund Code: 244631**<br>**Fund Title: FRQ-S/RSBO/UdeMtl**<br>**Current Year Award: $20,000.00**	**Local FST Contact:** Testa/Rocchina<br>rocchina.testa@mcgill.ca
**Project Title: Network for Oral and Bone Health Research (RSDO) - Recruitment Aid Program - 2016-2017 to support the conduct of the multicenter study "Efficacy of palatal brushing in patients with denture stomatisis: A randomised controlled tria"**	**RFMS Contact:** Suzanne Owsiany<br>suzanne.owsiany@mcgill.ca<br>**Faculty/Dept. Contact:** Rosemary Cooke
**Organization Code: 00023 - Dentistry**	**General Queries may be sent to the RFMS Help Desk:**<br>RFMSHELPDESK.FINSERV@MCGILL.CA

As Fund Holder, you automatically receive full access privileges to your fund in Minerva. Please consult and familiarize yourself with the Fund Financial Manager regulation at **http://www.mcgill.ca/financialservices/policies/ffm** for a description of the roles and responsibilities associated with this entitlement.

Information on policies and procedures relating to the financial management of your research fund can be found on the Financial Services Website - For Researchers tab at **http://www.mcgill.ca/financialservices/researchers**

Minerva:
A web interface to McGill's database, where all student, employee and financial data is stored. As a McGill faculty or staff member, you have access to a variety of self-service and administration menus, depending on your role in the McGill community. Minerva is accessible via the Web >> **http://www.mcgill.ca/minerva**

Use the Minerva self-service **Finance (Fund) Administration** menu to:

- Select **Budget Query for Fund Holders** for an up-to-the-minute fund balances and transaction details.
- Select **Financial Statements** to access your official monthly financial statement, available in PDF and SLK format, to save or print as desired.

Fund Balances and Profile Report:
A comprehensive financial summary of all funding sources (research/non research) was developed and tailored specifically for Principal Investigators (PIs) and Fund Financial Managers (FFM). The Fund Balances and Profile Report is an easy tool that is web based and can be run anytime, anywhere. You can use this tool to easily track the daily activities and balances for all your funds. Refer to the following link: **http://www.mcgill.ca/financialservices/researchers/querying**.

Delegation of Signing Authority and Approvals:
There are several policies/regulations which document when it is appropriate for a Fund Financial Manager (FFM), Principal Investigator (PI), or Unit Head to delegate their signing authority. Please refer to the following link for clear directives on delegation of signing authority and approvals: **http://www.mcgill.ca/financialservices/policies/delegation.**

Document Retention:
You may have documentation and retention responsibilities when it comes to transactions processed on your grants. Please refer to the following link for details relating to document retention: **http://www.mcgill.ca/financialservices/researchers**

Other Helpful Information:

FST: Trained staff who are locally situated in each faculty to assist you with the management of your research grants/contracts. See your FST contact name in the contacts area above. If you do not have a contact, please contact your RFMS Fund Administrator - Refer to contacts area above.

Need Minerva training? Contact the **Finance Help Desk at 398-3463**.

If you would like another faculty or staff member to query your fund, please complete the FIS Authorization form found at **http://www.mcgill.ca/financialservices/forms/fis** and fax it to **398-1326**.

We are here to assist you! Please contact your local FST representative (names in contact area above) to go over any aspects of your new research grant/contract or contact your RFMS Financial Administrator. The links in blue will lead you directly to referenced sites. We take this opportunity to extend our best wishes as you carry out your research activities.

Sincerely,
Elvie Coletta
Director

cc: Paul J Allison
