# Supplementary material for: Improving practice guidelines for the treatment of denture-related erythematous stomatitis: a study protocol for a randomized controlled trial
Source: Trials. 2017 May 5;18:211. doi: 10.1186/s13063-017-1947-y (PMC5420092; doi:10.1186/s13063-017-1947-y)
Supplement: Supplementary file 4 — Presents the ethical approval documents in English. (PDF 476 kb) [file 13063_2017_1947_MOESM4_ESM.pdf]

Université de Montréal

Comité d'éthique de la recherche en santé (CERES)

January 27th, 2017

BY EMAIL

Mr. Muhammad Faheem Khiyani,
Ph. D. (candidate) Biomedical sciences (médecine expérimentale)
Faculté de médecine dentaire - Département de dentisterie de restauration

Object : Reasearch ethics approval – Project « Efficacy of palatal brushing in patients with denture stomatisis: A randomised controlled trial » (certificate #16-003-CERES-D)

Dear Mr Muhammad Faheem Khiyani,

The purpose of this letter is to officially state that the enclose "Certificat d'éthique de la recherche" number #16-003-CERES-D was delivered for your project titled "Efficacy of palatal brushing in patients with denture stomatisis: A randomised controlled trial " funded by the CIHR. This research project is conducted under the supervision of Elham Emami & Jean Barbeau.

All the components submitted for approval were studied by the Health sciences research ethics board (Comité d'éthique de la recherche en santé). After you complied to the norms and rules of research ethics with human participants followed by the Université de Montréal, this certificate was delivered.

I also confirm that you make an annual follow-up on your project in order to maintain its validity.

Guillaume Paré
Research ethics advisor

adresse postale
C.P. 6128, succ. Centre-ville
Montréal QC H3C 3J7

3744 Jean-Brillant
4e étage, bur. 430-11
Montréal QC H3T 1P1

Téléphone : 514-343-6111 poste 2604
ceres@umontreal.ca
www.ceres.umontreal.ca

Université de Montréal
Comité d'éthique de la recherche en santé

7 March 2016

Objet: Approbation éthique – « Efficacy of palatal brushing in patients with denture stomatisis: A randomised controlled trial »

M. Muhammad Faheem Khiyani,

Le Comité d'éthique de la recherche en santé (CERES) a étudié le projet de recherche susmentionné et a délivré le certificat d'éthique demandé suite à la satisfaction des exigences précédemment émises. Vous trouverez ci-joint une copie numérisée de votre certificat; copie également envoyée à votre directeur/directrice de recherche et à la technicienne en gestion de dossiers étudiants (TGDE) de votre département.

Notez qu'il y apparaît une mention relative à un suivi annuel et que le certificat comporte une date de fin de validité. En effet, afin de répondre aux exigences éthiques en vigueur au Canada et à l'Université de Montréal, nous devons exercer un suivi annuel auprès des chercheurs et étudiants-chercheurs.

De manière à rendre ce processus le plus simple possible et afin d'en tirer pour tous le plus grand profit, nous avons élaboré un court questionnaire qui vous permettra à la fois de satisfaire aux exigences du suivi et de nous faire part de vos commentaires et de vos besoins en matière d'éthique en cours de recherche. Ce questionnaire de suivi devra être rempli annuellement jusqu'à la fin du projet et pourra nous être retourné par courriel. La validité de l'approbation éthique est conditionnelle à ce suivi. Sur réception du dernier rapport de suivi en fin de projet, votre dossier sera clos.

Il est entendu que cela ne modifie en rien l'obligation pour le chercheur, tel qu'indiqué sur le certificat d'éthique, de signaler au CERES tout incident grave dès qu'il survient ou de lui faire part de tout changement anticipé au protocole de recherche.

Nous vous prions d'agréer, Monsieur, l'expression de nos sentiments les meilleurs,

Dominique Langelier, présidente
Comité d'éthique de la recherche en santé (CERES)
Université de Montréal

DL/GP/gp
c.c. Gestion des certificats, BRDV
Elham Emami, professeure agrégée, Faculté de médecine dentaire
Jean Barbeau, professeure titulaire, Faculté de médecine dentaire -Département de stomatologie
Suzanne Valiquette
p.j. Certificat #16-003-CERES-D

adresse postale
C.P. 6128, succ. Centre-ville
Montréal QC H3C 3J7

3744 Jean-Brillant
4e étage, bur. 430-11
Montréal QC H3T 1P1

Téléphone : 514-343-6111 poste 2604
ceres@umontreal.ca
www.ceres.umontreal.ca

Université de Montréal

N[O] de certificat
16-003-CERES-D

Comité d'éthique de la recherche en santé

CERTIFICAT D'APPROBATION ÉTHIQUE

Le Comité d'éthique de la recherche en santé (CERES), selon les procédures en vigueur, en vertu des documents qui lui ont été fournis, a examiné le projet de recherche suivant et conclu qu'il respecte les règles d'éthique énoncées dans la Politique sur la recherche avec des êtres humains *de l'Université de Montréal.*

**Projet**	
**Titre du projet**	**Efficacy of palatal brushing in patients with denture stomatisis: A randomised controlled trial**
**Étudiant requérant**	**Muhammad Faheem Khiyani** (ND), Candidat au Ph. D. Sciences biomédicales (médecine expérimentale), Faculté de médecine dentaire - Département de dentisterie de restauration
Sous la direction de	Elham Emami, professeure agrégée, Faculté de médecine dentaire, Université de Montréal & Jean Barbeau, professeure titulaire, Faculté de médecine dentaire -Département de stomatologie, Université de Montréal.
Autres membres de l'équipe:	Jocelyne Feine (McGill University), codirectrice.
**Financement**	
Organisme	IRSC
Programme	
Titre de l'octroi si différent	
Numéro d'octroi	
Chercheur principal	
No de compte	

MODALITÉS D'APPLICATION

Tout changement anticipé au protocole de recherche doit être communiqué au CERES qui en évaluera l'impact au chapitre de l'éthique.

Toute interruption prématurée du projet ou tout incident grave doit être immédiatement signalé au CERES

Selon les règles universitaires en vigueur, un suivi annuel est minimalement exigé pour maintenir la validité de la présente approbation éthique, et ce, jusqu'à la fin du projet. Le questionnaire de suivi est disponible sur la page web du CERES.

Dominique Langelier, présidente<br>Comité d'éthique de la recherche en santé<br>Université de Montréal	**7 mars 2016**<br>Date de délivrance	**1er janvier 2018**<br>Date de fin de validité

adresse postale
C.P. 6128, succ. Centre-ville
Montréal QC H3C 3J7

3744 Jean-Brillant
4e étage, bur. 430-11
Montréal QC H3T 1P1

Téléphone : 514-343-6111 poste 2604
ceres@umontreal.ca
www.ceres.umontreal.ca

Atest.CEP 001/2017

ATESTADO

Atestamos que o Protocolo nº CAAE 37033814.60000.54190 sobre a pesquisa intitulada **"O efeito da escovação do palato sobre a estomatite relacionada à prótese: um ensaio clínico randomizado"**, sob a responsabilidade do Prof. Dr. Raphael Freitas de Souza, está de acordo com os Princípios Éticos adotados pelo Comitê de Ética em Pesquisa da Faculdade de Odontologia de Ribeirão Preto, USP, foi APROVADO em 08/12/2014.

We certify that the protocol nº CAAE 37033814.60000.54190 about the research entitled **""The effect of palatal brushing on denture-related stomatitis: a randomized controlled trial"**, under responsibility of Prof. Dr. Raphael Freitas de Souza, is in accordance with the Ethical principles adopted by the Research Ethics Committee of the School of Dentistry of Ribeirão Preto, University of São Paulo, Brazil, was approved in 08/12/2014.

Ribeirão Preto, 25 de janeiro de 2017.

Profa. Dra. Simone Cecílio Hallak Regalo
Coordenadora do Comitê de Ética em Pesquisa

UNIVERSIDAD DE LA FRONTERA

COMITÉ ÉTICO CIENTÍFICO

Report N°: N°043/2015

ASSESSMENT REPORT
RESEARCH PROTOCOL FOLIO Nº013/15

The Scientific Ethics Committee of the Universidad de La Frontera in Temuco, (Exempt Resolution No. 1090 dated March 12, 2014), in ordinary meeting No. 08 dated 27 May 2015, chaired by Dr. Claudia Barchiesi Ferrari, with the assistance of its permanent members: Fernando Borie Borie, Cantín Mario López, Jaqueline Caniguan Caniguan, Roberto Contreras Eddinger, Mauricio García Ojeda, Jaime Guerrero Contreras, Horacio Miranda Vargas, Karin Morales Manríquez, Mónica Pineda Nesbet, Sergio Salgado Salgado, Jaime Tijmes Ihl, Julio Valdés Autonell, report evaluated and sanctioned the research protocol:

"Efficacy of palatal brushing in patients with denture stomatitis: a randomized clinical trial"

Principal Researcher: Ph.D Ramón Fuentes Fernández.

Para constancia firman:

SRA. MÓNICA PINEDA NESBET
EXECUTIVE SECRETARY
SCIENTIFIC ETHIS COMMITTEE
UNIVERSIDAD DE LA FRONTERA

DR. MARIANO DEL SOL CALDERÓN
PRESIDENT
SCIENTIFIC ETHICS COMMITTEE
UNIVERSIDAD DE LA FRONTERA

Temuco, January 16, 2017

cc.
Ph.D. Ramón Fuentes Fernández, Principal Researcher
Scientific Ethics Committee file.

McGill

Faculty of Medicine
3655 Promenade Sir William Osler #633
Montreal, QC H3G 1Y6

Faculté de médecine
3655, Promenade Sir William Osler #633
Montréal, QC H3G 1Y6

Fax/Télécopieur: (514) 398-3870
Tél/Tel: (514) 398-3124

20 June 2016

Dr. Raphael F. de Souza
Faculty of Dentistry
2001, avenue McGill-College, Suite 534
Montreal QC H3A 1G1

RE: IRB Review Number A00-M25-16A
Efficacy of palatal brushing in patients with denture stomatitis: a randomized controlled trial

Dear Dr. de Souza

On 13 June 2016, at a meeting of the Institutional Review Board, a full Board review was conducted for the above-referenced study.

The Committee identified the following concerns for your response:

Scientific Protocol & Ethical Considerations

1. How will adherence to palatal brushing be assessed? The Committee recommends having participants record daily brushing in a tracking diary.
2. What mechanisms are in place to maintain the study blind (i.e. to keep participants from self-declaring to their dentist?)
3. Questionnaires/Data Collection Forms:

a. Assessment of Oral Condition – the committee recommends replacing the term 'prosthesis' with 'dentures' throughout all forms provided to participants.
b. Correct the spelling of 'Sociodemographic Information' on the English form.
c. Lifestyle – question 9 – recommend revising the parenthetical text to read, '(number of drinks per day, or approximate volume consumed in either ounces or mL.)
d. Complete prosthesis – question 1 – define 'edentulous'.

Recruitment Ad/Poster

4. In the third sentence, replace '…in reducing inflammation...' with '…on reducing inflammation…'
5. Define or rephrase 'xerostomia'.

Consent Form

6. The English and French consent forms do not contain the same information; the two forms need

to be consistent.

7. The English translation would benefit from a review by a writer fluent in the English language to correct grammar and sentence structure.
8. Correct the page number on the French consent form.
9. Under *General Information* – replace 'disadvantages and advantages' with 'risks and benefits'.
10. Delete the section 'Conditions to participate'; listing the eligibility criteria is not a requirement for the consent form.
11. Under *Nature of participation and duration of the study* – please provide a brief explanation for 'ultrasonic back'.
12. The procedures and associated risks sections provide no information on the collection of samples for biomarker/genetics testing (i.e. what the saliva samples will be used for and why.) Additionally, please clarify whether this testing is mandatory or an optional component to study participation. If it is optional, then an explicit and separate consent for this testing must be obtained from the study participant.
13. Under *Associated risks and inconveniences* – in the last sentence of the first paragraph, replace the word 'drawbacks' with 'inconveniences' and rephrase the end of this sentence to read, '…associated with participating in this research project.'
14. Modify the 'Advantages and benefits' section header to simply read 'Benefits', and revise the text to read: 'You may or may not benefit personally from taking part in this project. One potential benefit is a possible reduction in the inflammation of your palate. You will also receive the required brushes free of charge. You will benefit from having your dentures cleaned with ultrasound technology at each study visit. This study will provide researchers with new information about the impact of palatal brushing in denture wearers, and the results of this study may help in the development of better treatment for denture stomatitis.'
15. Under *Confidentiality* – modify the end of the first sentence to read, '…to meet the project's scientific objectives.'
16. Under *Confidentiality* - add the following statement, 'Members of the McGill Institutional Review Board, or persons designated by this Board, may access the study data to verify the ethical conduct of this study.'
17. On the English consent form, replace the term 'Results diffusion' with 'Communication of results'. Additionally, provide participants with the option to receive the results by mail if they do not use or do not have access to email or the internet.
18. Under *Voluntary participation and withdrawal* – delete 'or exclusion' from the last sentence of the second paragraph.
19. Delete the section 'Registration of clinical trial'; this is a US requirement for regulated clinical trials and does not require statement in consent forms for studies conducted in Canada.
20. Move the section 'interruption of clinical trial' to follow the section on voluntary participation and withdrawal. Additionally, change the section header to read, 'Discontinuation of the study'.
21. Replace the section title 'Identification of contact persons' with 'Contact information for questions', and clearly separate the name and contact information for questions about the research study and for questions about the rights of research participants.
22. Consent Form/Signature page: rewrite the consent statement as follows: 'The research project described in this consent form has been explained to me. I am aware of the purpose of this study, what I am asked to do, and the risks and benefits of taking part. Any questions I had about this study have been answered, and I can obtain more information at any time during the study. I am aware that I can withdraw from this study at any time. I consent to take part in this study. I do not give up any of my legal rights by signing this consent form. I will receive a copy of this

consent form for my records.'

23. Consent Form/Signature page: replace the French text with English (i.e. Name of Participant (printed), Signature of Participant.)

This study received ethics approval pending the submission and an assessment of an appropriate response to the above-outlined issues.

Regards,

Roberta Palmour, PhD
Chair
Institutional Review Board

Cc: A00-M25-16A
